# The dynamic nature of parenting practices: a qualitative enquiry of parenting adolescents during COVID-19

**DOI:** 10.3389/fpsyg.2024.1309786

**Published:** 2024-04-22

**Authors:** Nandita Babu, Mehreen Fatima, Manushi Arora

**Affiliations:** Department of Psychology, University of Delhi, New Delhi, India

**Keywords:** parenting, adolescents, pandemic, COVID-19, dynamic nature

## Abstract

**Introduction:**

Parenting practices are known to be dynamic, evolving in response to various factors such as societal changes, cultural norms, and individual circumstances. Understanding the dynamic nature of parenting is essential for comprehending its influence on children’s development. This study aimed to explore the adaptive nature of parenting practices amidst the backdrop of the COVID-19 pandemic, a global event that provided a unique context for examining these dynamics.

**Methods:**

In-depth semi-structured qualitative interviews were conducted with six heterogeneous couples who were parenting at least one adolescent during the pandemic. Attride-Sterling’s thematic network analysis was employed to analyze the interview data.

**Results:**

Five global themes emerged from the data, namely, change in parenting style due to stressors, paternal versus maternal style of parenting, intergenerational transmission of parenting practices, child-centric parenting practices, positive parenting practices and evolving parent–child relationship during COVID-19.

**Discussion:**

The findings highlight the tendency of parents to adapt their parenting styles to meet the evolving needs of their children. Understanding these dynamics is crucial for supporting families during times of crisis. Future research should explore the long-term effects of these changes and effective strategies for promoting positive parent-child relationships.

## 1 Introduction

Parenting is essentially an indispensable aspect of child rearing and has gained considerable attention from the diverse disciplines over the past 75 years, owing to the crucial role that it plays in child development (Kuppens and Ceulemans, 2019). It is a complex activity that involves many specific observable behaviors, for instance, reward and punishment, limit setting, reasoning, etc., which parents utilize to socialize their children (Darling and Steinberg, 1993). Parenting practices are considered to be fundamental to a child’s growth, particularly for the development of their social competencies, emotional adjustments, and academic achievements (Baumrind, 1991; Maccoby, 2000; Patterson and Fisher, 2002; Bornstein and Bornstein, 2007).

The quality of parenting is one of the modifiable factors that can influence the course of the child’s development (Collins et al., 2000). Maccoby and Martin (1983) found that the two key qualitative dimensions of parenting that have positive outcomes for children include warmth/affection and parental control. For instance, it is undeniable that a “well parent” is a pre-requisite to parenting well (Smith, 2010). A parent who is stressed or depressed himself/herself is unlikely to be responsive to the needs of their children and faulty parenting practices have often been cited as the major causal factor in child psychopathology (Gelfand and Teti, 1990; Goodman, 2003; Newman and Stevenson, 2005). Moreover, an individual’s parenting is influenced by their own experiences of being parented and the attitude and expectations they bring to it (Bornstein, 2002).

The parents tend to have varied approaches concerning how they interact and guide their children, through which the children’s morals, principles, and conducts are established (Sanvictores and Mendez, 2021). Over the past few decades, a great deal of research has focused on the typological approach toward parenting, which was originally propounded by Baumrind (1971) and later expanded upon by Maccoby and Martin (1983). The four major types of parenting styles include authoritarian parenting, authoritative parenting, permissive parenting, and uninvolved parenting style, all of which utilize a unique approach to raising children.

The authoritarian parenting style is characterized by a one-way mode of communication in which the parents establish strict rules that the child is expected to follow. Failure to adhere to these rules usually leads to punishment, as there is little to no scope for flexibility. Authoritative parenting involves a close nurturing relationship between parents and children, in which parents motivate verbal engagements, indulge in disciplinary actions but also explain the rationale behind them, and employ efficient practices to reinforce appropriate behavior. In permissive parenting, the parents are usually warm and nurturing, and have no expectations from their children, which leads to the rare use of discipline. Parents who employ this style tend to be extremely affirmative toward the child’s desires and actions, and children are also consulted in important family decisions. Finally, the uninvolved parenting style is considered to be the most detrimental form of parenting, in which parents fail to monitor the behavior of their children and are also unresponsive to most of their needs (Baumrind et al., 2010).

The typological approach to parenting may give an impression that the parenting style of an individual is inflexible and remains static throughout, however, most parents do not follow or adhere to a single model completely. Shahsavari (2012) argues that cultural, ethnic, social, political, and economic factors can influence the parenting style, which may influence the specific parenting behaviors. The individual and contextual factors that influence parenting have been explicated by many theoretical frameworks, for instance, Belsky’s process model of the “determinants of parenting” (Belsky, 1984), which explains how parenting is influenced by the characteristics of the child, the parent, as well as their social context; Abidin’s model of parenting stress (Abidin, 1992), which proposes that high levels of parental distress, child maladjustments, and parent–child dysfunctional interactions often lead to negative parenting, which in turn has a negative influence on the child’s behavior.

At a broader level, child-rearing practices tend to be influenced by one’s culture (Chen et al., 2019), socio-economic status (Hoff and Laursen, 2019), and neighborhood/community (Kotchick and Forehand, 2002). In a cross-sectional study examining the generational changes in parenting styles and the effect of culture on parenting, Zervides and Knowles (2007) found that a generational change from authoritative to a more lenient and democratic style of parenting has occurred and that Greek-Australian parents reported more authoritarian child rearing style than the Anglo-Australian counterparts. Furthermore, as a corollary, an individual’s own parenting style may also change from time to time. For instance, the parenting style changes from one child to the next, and sometimes, the circumstances in the lives of a parent may also influence their parenting style. The parents may deal with their children in an authoritarian way when they are tired but in an authoritative manner when they feel energetic. As such, parenting is not a static entity, rather it is shaped by a variety of factors that are themselves changing over time (Belsky, 1984).

The Covid-19 pandemic that began in 2019 has emerged as a novel context, which has instigated fundamental changes in the family system. The lengthened social and physical isolations that were inherent to covid-19 notably disrupted the family routine. A cross-sectional study in northeastern US on 2,068 parents revealed that exposure to covid-19 related stressors and distress uniquely predicted the parental use of neglect, and use of harsh and positive parenting practices (Connell and Strambler, 2021). Wolf et al. (2021), in their research on 342 parents residing in Central Ohio, found that higher levels of parental stress were associated with greater odds of punitive parenting. Yet another research by Wissemann et al. (2021) on 87 US participants having at least one child at home found that the negative emotions, such as fear, anger, sadness, and loneliness were associated with the controlling parenting behaviors.

Particularly, parenting adolescents during a pandemic can be a taxing task, given the myriad of changes that characterize adolescent years. Adolescence is a stage between childhood and adulthood in which individuals go through rapid physical, cognitive, and psychosocial development. Parenting an adolescent is considered to be challenging as parents are required to continually adapt their parenting strategies to the changing motivations and demands of this stage, wherein an adolescent is continually seeking autonomy and is increasingly asserting their independence (Soenens et al., 2019). These changes, in addition to the major stressors hurled by the pandemic, can emerge as a novel context to understand the dynamic nature of parenting.

However, despite having awareness of parenting as a dynamic process, limited research has attempted to document the “how” and “why” of the changes in parenting behaviors (Strohschein et al., 2008). Therefore, the present study aimed to explore the dynamic nature of parenting practices within the broader context of family dynamics. While the COVID-19 pandemic served as the contextual framework due to its novel and impactful nature, it is important to note that the study primarily focused on elucidating the dynamics of parenting strategies and behaviors. Moreover, the research aimed to shed light on the factors that influence changes in parenting practices over time, irrespective of the specific contextual backdrop. By examining the interplay between individual, familial, and contextual factors in shaping parental behaviors, the study seeks to identify underlying mechanisms that contribute to the ongoing evolution of parenting strategies. The adolescent population was chosen for the present study as it is a challenging stage of life during which an individual is going through significant changes and the parenting practices employed during adolescence continue to affect their behavior later into adulthood (Hoskins, 2014), thus making the adolescent-parent relationship a crucial factor to scrutinize in the human development.

## 2 Method

### 2.1 Sample description

The sample consisted of 6 heterosexual couples, i.e., 12 participants in the age range of 39 to 53 years, who were parenting at least one adolescent in the age range of 12 to 18 years. The participants were selected through convenience sampling procedure, keeping in view the restrictions placed during pandemic. The educational qualification of the participants ranged from completing 12th to masters and M. Ed., and largely belonged to financially well-off families residing in Delhi-NCR, India. In addition, three out of six male participants were businessmen whereas four out of six female participants were homemakers. Full demographic details of the participants along with the pseudonyms are given in Table 1.

**Table 1:** Parent participant characteristics.

Pseudonym	Gender	Age	Educational qualification	Occupation	Children
Mr. A	Male	53 years	Masters	IRS officer (civil servant)	17-year-old and 21-year-old daughters
Mrs. A	Female	43 years	Graduation	Homemaker	
Mr. B	Male	50 years	B. Tech.	Mechanical Engineer	16-year-old daughter (single child)
Mrs. B	Female	40 years	Graduation	Homemaker	
Mr. C	Male	47 years	Intermediate	Businessman	14-year-old and 7-year-old daughters
Mrs. C	Female	39 years	Graduation	Businesswoman	
Mr. D	Male	50 years	Intermediate	Businessman	16-year-old and 20-Year-old sons
Mrs. D	Female	43 years	Graduation	Homemaker	
Mr. E	Male	44 years	Graduation	Homemaker	16-year-old and 8-year-old daughters
Mrs. E	Female	45 years	Intermediate	Service in MNC	
Mr. F	Male	45 years	Intermediate	Businessman	13-year-old daughter and 8-year-old son
Mrs. F	Female	40 years	Masters, M. Ed.	Homemaker	

### 2.2 Data collection and procedure

Prior to data collection, all subjects gave their written informed consent for inclusion in the study. The study was conducted in accordance with the Declaration of Helsinki (1964) and its later amendments, and the protocol was approved by the (masked for review). The data was collected with the help of in-depth semi-structured interviews consisting of both open-ended as well as leading questions, which lasted for about 30–45 min. All the interviews were audio-recorded after obtaining due consent of all the participants and were later transcribed to ensure a reliable analysis. Further, hand written notes were also taken at the time of the interviews for the observations made, so as to comprehend the participants’ view about the concerned issue, to make sense of the data, and to ease the process of organizing them into categories and themes at the time of analysis. The semi-structured interview schedule is attached in the electronic Appendix.

### 2.3 Tool of data analysis

The data was analyzed using the “*Thematic Network Analysis*” by Attride-Stirling (2001). It is a qualitative method that involves organizing themes at different levels, wherein these networks aim at structuring the data depicting the themes in a representational form. These networks offer a web-like structure as an organizing principle as well as representational means, defining the course of actions that would be used in moving from text to interpretation. Thematic Network Analysis involves moving from Basic Themes toward a Global Theme, by combining the Basic Themes into Organizing Themes on the basis of the underlying stories that they are telling. Organizing Themes are then reinterpreted in the light of Basic Themes and are clubbed together to represent an over-arching conclusion in the form of a Global Theme.

## 3 Results

The five global themes were identified, incorporating the sub-themes, organizing themes, and basic themes, after coding each of the 12 interviews individually and thematically analyzing them. The five global themes that emerged after carefully analysing the data are described as ahead.

### 3.1 Change in parenting style due to stressors

The first global theme pertains to the different sources of stress that arose at the time of pandemic situation, which had an impact on the child rearing practices. Some of the sources of stress identified by the parents that impacted their parenting are financial stressors, lockdown related stressors, and stress arising out of conflict between couples. For instance, one of the participants was quoted as saying:

*“maine bataya toh irritation, frustration, mujhe gussa itna aane lag gya hai, patience chali gai hai. Jaise main bahut loud hojaati hu fir mujhe thodi der baad realize hota hai ki nahi bolna, chup rehna hai… I think yeh jo puura cycle hai shout karna fir chodh dena, yeh kaafi badhgaya pandemic main pehle jo mahine main 1–2 baar hota tha lagta tha jab nahi handle horaha ab voh hafte main 2–3 baar hota hai,”* translating to “as I told earlier, …irritation, frustration, I have started getting too angry, have lost all patience… for example I end up getting really loud and then I realize I should have stayed quiet… I think this whole cycle of getting angry and then leaving it all, this has increased during the pandemic, it now happens around 2–3 times a week although it was only 1–2 times a month earlier” (Mrs. A).

Yet another participant was quoted as saying:

*“baki main apne stress main hi rehta tha zyada time nhi spend kar pata tha meri wife hi dekhti hi inhe.,”* translating to “I used to remain stressed (due to the pandemic), wasn’t able to spend enough time with the kids, and so my wife used to look after them” (Mr. A).

Another verbatim sheds light on the marital clashes owing to the pandemic leading to the effect on parenting:

“*woh mera gussa baccho par nikaal dete hai”; “ghar par itni ladayi badh gayi ha*i,” translating to “he displaces my anger onto the children”; “there has been an increase in fights at home” (Mrs. D).

### 3.2 Paternal versus maternal style of parenting

The second global theme is concerned with delineating the differences between the father’s and mother’s style of parenting in raising their children. The theme indicated that whereas fathers had a surface level engagement with children owing to the time constraints, mothers played a more active role in raising children, which was acknowledged by fathers as well. As one of the female participants, i.e., mother, was quoted as saying:

*“Papa ka as such itna involvement nahi hai unko lagta hai padhle, jitna marks le aaye, effort kare bas as such isse zyada kuch nahi. Voh jitna financially support kar sakte hai karte hai…,”* translating to: “as such, the father has never remained too much involved… for him, whatever marks kids get is fine for as long as they are working hard… he does provide financial support to the extent possible…” (Mrs. C).

Another verbatim exemplifies the father’s style of parenting:

*“Jab aakar baatein karlete hai main beth jaata hun aise unavailable nahi rehta but mujhe waise itna kaam hota hai main kya kya manage karun?,”* translating to: when they come to talk, I sit down… it is not like I’m unavailable… but I have so much work to do, what all should I manage?” (Mr. F).

### 3.3 Intergenerational transmission of parenting practices

The third global theme is concerned with the process through which the previous generation tends to influence the parenting behavior of the present generation, either deliberately or unintentionally… Some of the organizing themes under this global theme were-child rearing practices as different from one’s own parents, and replicating child-rearing practices from one’s own parents. While reflecting on their own parenting styles, the parents shed light on the parenting tactics that they carried forward from their own parents and the ones that they modified to suit the present times. For instance, one of the participants reflected on the adaptive nature of intergenerational parenting:

“*I share my personal experiences with her too, I tell her how I used to go to my mom and tell her things but she would not always be there so I want to make sure I’m the first person she tells things to.*” (Mrs. A).

At the same time, she also reflected on how she retained the core values of her parents that she found valuable in the present times as well:

“*because mere mummi papa hamesha se padhai ko bahut importance dete the aur padhai mere liye bhi ek core value thi toh of course main apne baccho ko bhi voh karne ke liye zyada encourage karungi hi.*,” translating to- “because my parents used to give a lot of importance to education and hence education was a core value for me too… so I will also encourage my kids to study…” (Mrs. A).

Yet another participant reflected on how she did not carry forward the parenting practices that she found unsuitable:

*“Aur jaise maine use kabhi roka nahi woh karne se jo uska dil karta hai…. mera mummy gussa karti thi chid ke beth jati thi baat nahi karti thin mere se*… *ye maine iske sath kabhi nahi kia usko drawing karne ka shauk hai karo theek hai*.”:, translating to: and I never stopped her from doing what she wanted to do… my mother used to get angry and irritated, she would not talk to me (for following her passion)… I never did this with her… She has a hobby of drawing…(I was like) okay do it” (Mrs. F).

### 3.4 Child-centric parenting practices

The fourth global theme outlined the type of parenting that is centred around prioritizing the needs and interests of the child and changing the parenting style depending upon the factors inherent to the child. Some of the organizing themes under this global theme were- age-dependent parenting practices, gender-dependent parenting practices, birth-order and parenting practices, parenting practices influenced by the inherent nature of the child, etc. The child-centric parenting becomes evident in the following verbatims:

*“but haan meri beti ab 8th main ja rahi hai toh I try to give her that space jab voh irritate hoti hai ya shout karti hai mujhpar toh main usse space deti hu to relax and come back to me because I do not want her to drift away during her teenage years,”* translating to: “now that my daughter is in 8th standard, I try to give her space whenever she is irritated or shouts at me… for her to relax and come back to me because I do not want her to drift away during her teenage years” (Mrs. C).

*“I understand gussa karne se heated argument hota hai aur cheezei worsen ho jaati hai toh inke mood ke hisaab se smjhna padta hai,”* translating to: I understand that anger leads to heated arguments and worsen things, so we have to understand things as per their mood” (Mrs. A).

### 3.5 Positive parenting practices and evolving parent–child relationship during Covid-19

The fifth and final global theme outlined the positive changes in parenting practices owing to the pandemic. Many parents in their interviews reported that pandemic eventually emerged as an opportunity to rejuvenate oneself by engaging in family activities wherein the parents could make up for the lost time with their children by engaging in recreational activities such as cooking food, playing board games, watching movies, etc. by making conscious efforts to keep them constructively engaged during the pandemic. Some of the verbatims that elucidate this theme are:

*“ye corona ke andar hi time mila hai*… *toh ab pandemic main yehi hota tha ghar par rehte the sab baatein karlete the jo pehle nahi ho paata tha*.,” which translates to: “it is only during corona that we have got time… due to the pandemic, we all used to stay indoors, which gave us an opportunity to interact with each other, which wasn’t possible earlier” (Mr. A).

*“Main aur meri wife dono hi hum kaafi pyaar se deal karte the baccho se, kabhi zyaada gussa nahi karna*… *unse baatein share karna jo ki voh shayad nahi kar paaye time na hone ki wajah se. Aaj humare paas especially pandemic ki wajah se luxury hai time spend karne ki toh dhang se usse utilize karo na*…,” which translates to: “I and my wife used to deal very affectionately with the kids… never got too angry, even shared things that we could not earlier due to the lack of time. Now that we have the luxury of spending time with them especially due to the pandemic, why should not we utilize it?” (Mr. B).

The thematic networks for the five global themes have been outlined in the Appendix.

## 4 Discussion

**Figure 1:**
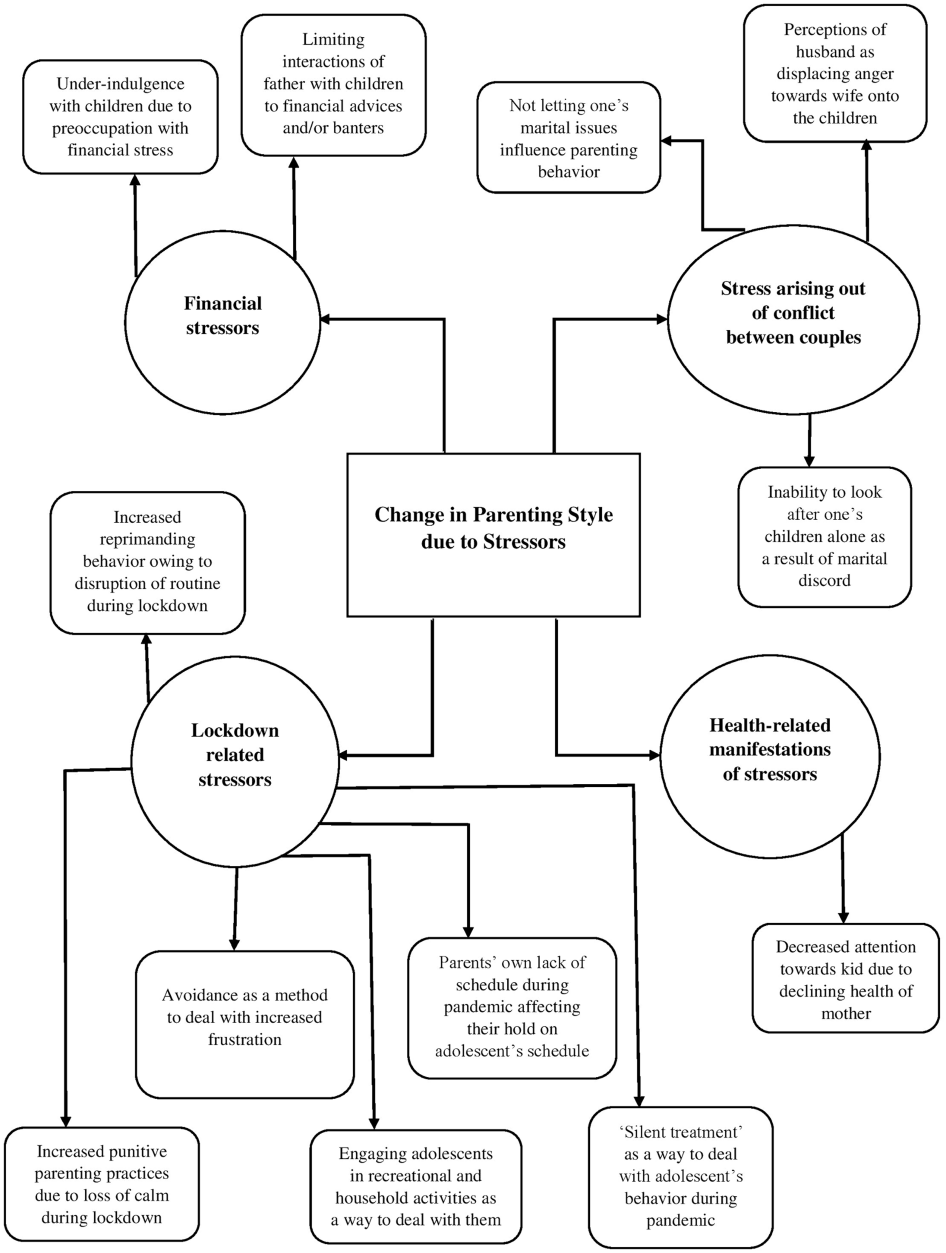
Represents the global theme “change in parenting style due to stressors”.

**Figure 2:**
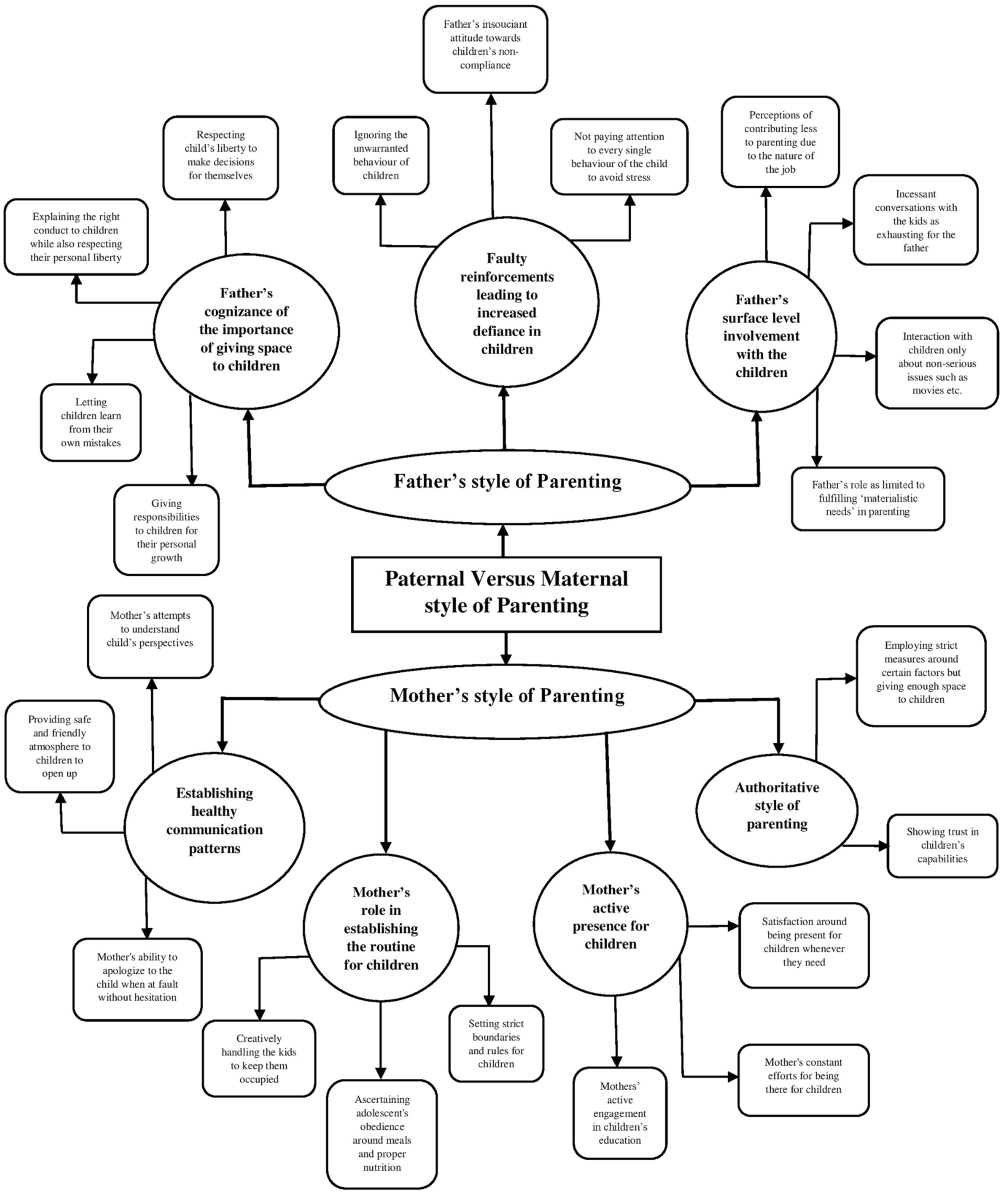
Represents the global theme “paternal versus maternal style of parenting”.

**Figure 3:**
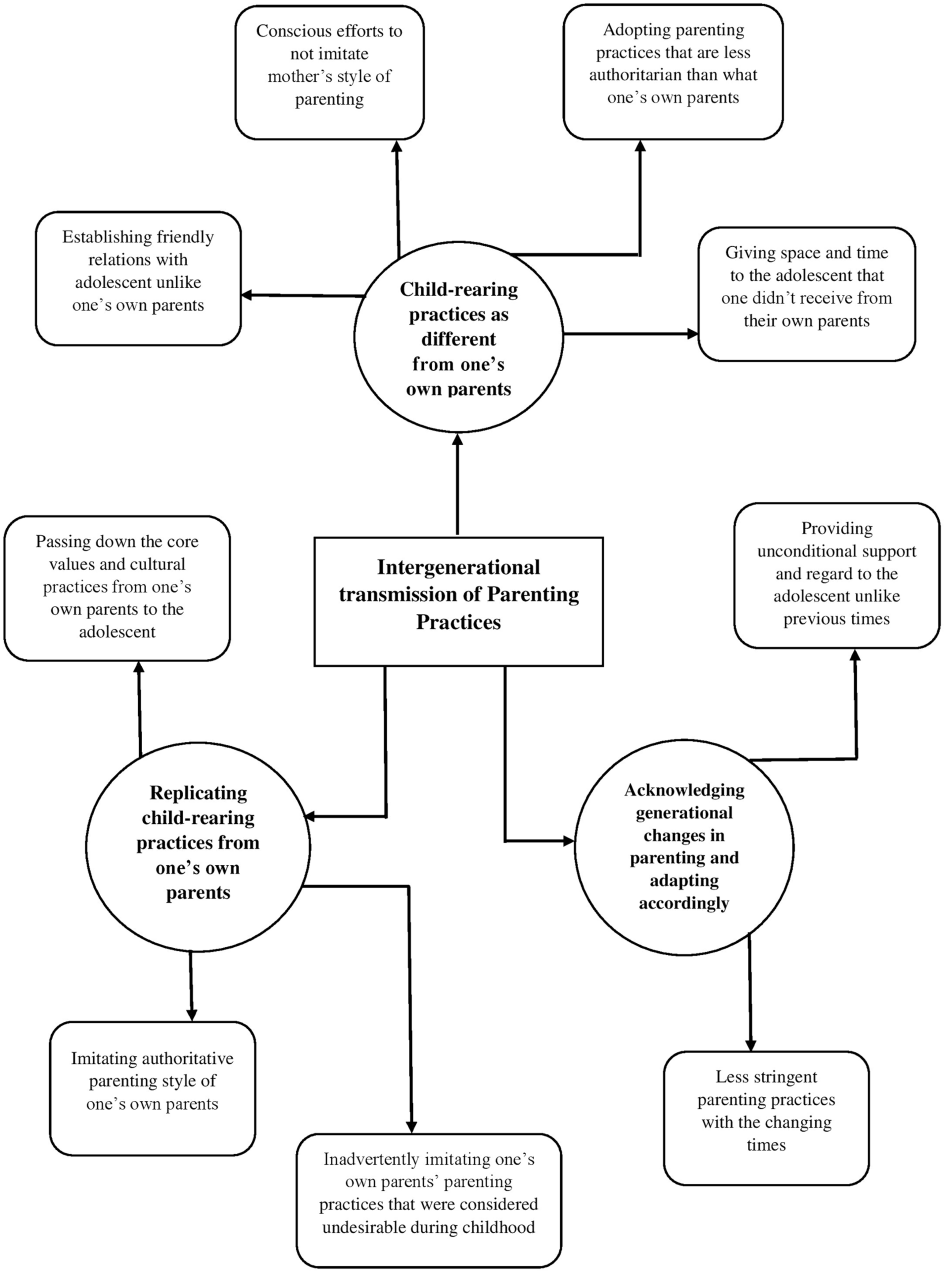
Represents the global theme “intergenerational transmission of parenting practices”.

**Figure 4:**
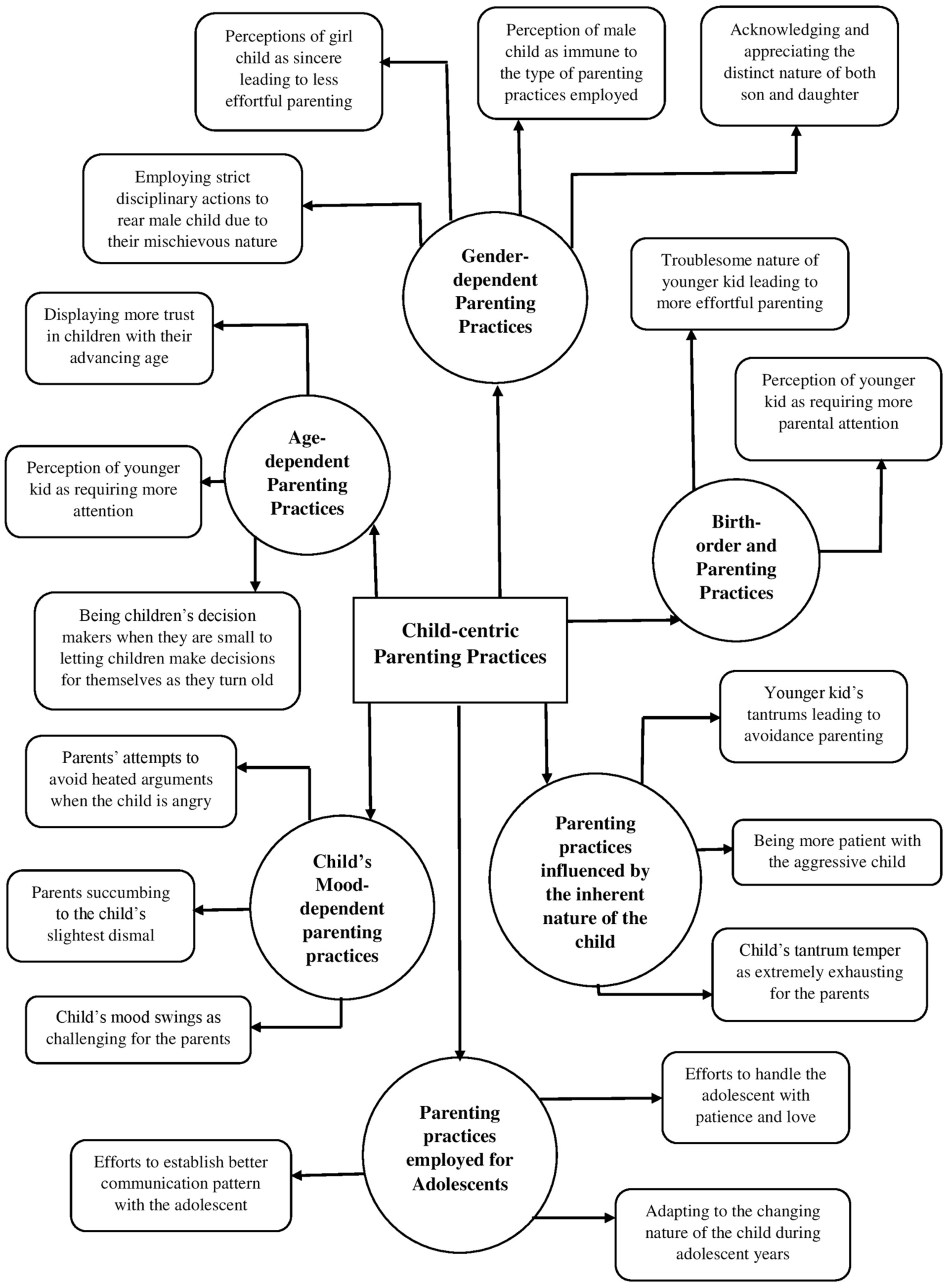
Represents the global theme “child-centric parenting practices”.

**Figure 5:**
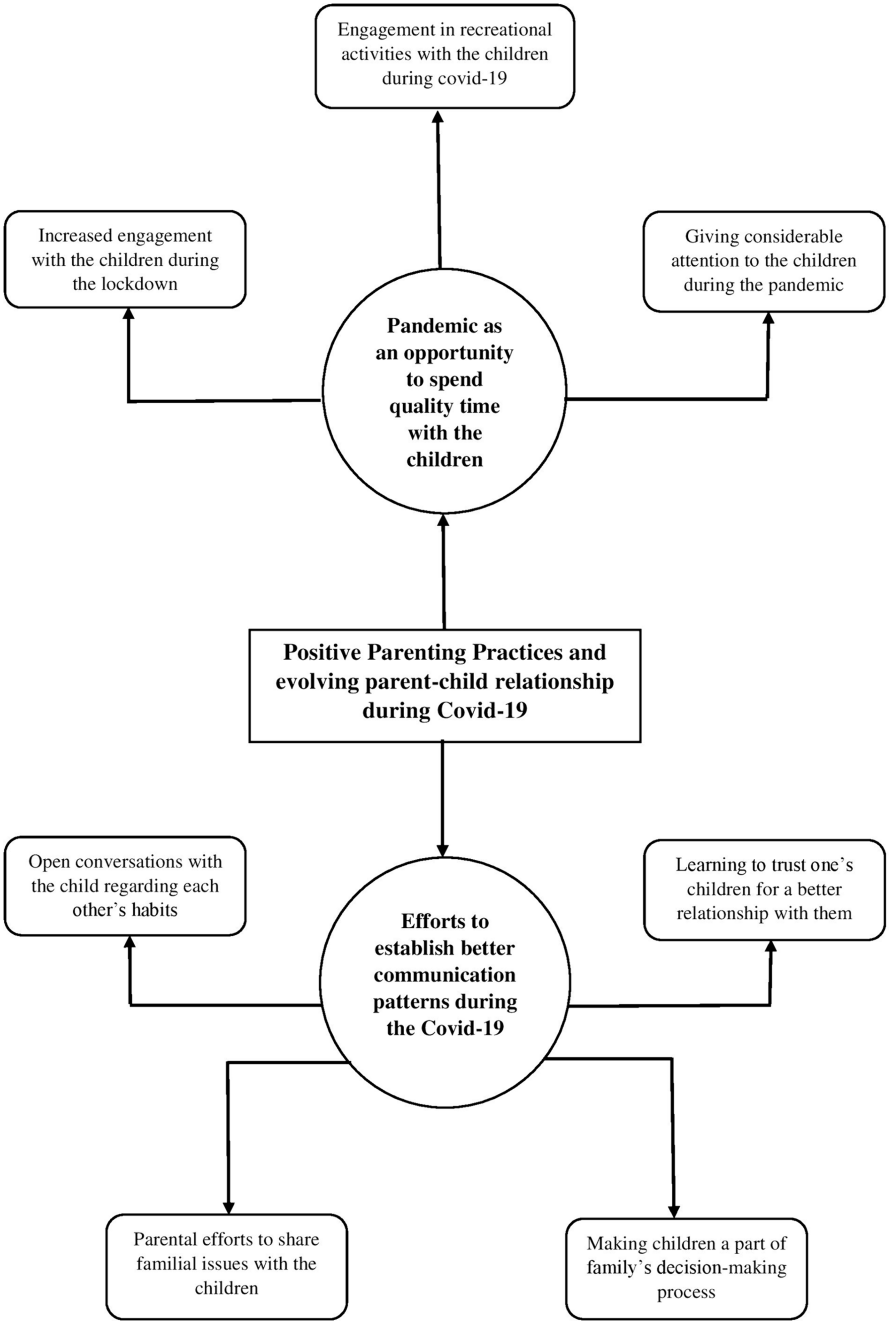
Represents the global theme “positive parenting practices and evolving parent–child relationship during Covid-19”.

Parenting is considered to be a lifelong endeavor that has consequences for the well-being of the children, particularly in terms of the child’s behavior and temperament (Smith, 2010). The present study aimed to explore the dynamic nature of parenting practices, taking the Covid-19 pandemic as the contextual framework. The thematic networks were derived from the interviews of six couples, by utilizing the ‘Thematic Network Analysis” approach (Attride-Stirling, 2001). The five global themes that were identified are- (i) change in parenting style due to stressors, (ii) paternal versus maternal style of parenting, (iii) intergenerational transmission of parenting practices, (iv) child-centric parenting practices, and (v) positive parenting practices and evolving parent–child relationship during the Covid-19. Our findings are emblematic of the dynamic nature of parenting practices based on several different characteristics as well as the underlying context, which has also been asserted by various other researchers (for e.g., Smetana, 2017; see Figures 1–5).

The first global theme shed light on how the different stressors that the parents are faced with, tend to spill over to their child-rearing practices. The initial emergent narratives in our study spun around the different sources of stress in the pandemic, leading to problematic parenting, either directly, or mediating through various other factors, such as declining health, and marital conflict, amongst others. The findings are supported by various other studies on parenting behavior during the Covid-19 pandemic. Chung et al. (2022), in their study on 258 participants residing in Singapore, found that the perceived impact of covid −19 and harsh parenting style was mediated by parental stress. Lev-Wiesel et al. (2022), in their study on 236 parents to children in the age range of 3-to-16 years also found that the parents who had lost their jobs during the pandemic had higher levels of depression and employed emotionally aggressive tactics to deal with their children. Indeed, there is a complex interplay between the parenting practices employed by the individuals and their effects on the child-rearing practices, which in turn has repercussions for the child’s behavior (Ward et al., 2015). For instance, research indicates that parental stress is an environmental risk factor that is both an antecedent to and has consequences for problematic child behavior (Neece et al., 2012).

The second global theme elucidated the differences between the father’s and mother’s styles of parenting in raising their children, which is considered to be a theoretically warranted phenomenon in child-rearing practices. In the present study, we found that the mothers and fathers had different roles, responsibilities, and parenting styles when it comes to raising their children. Indeed, mothers and fathers utilize different parenting styles and practices owing to the different roles that they play in the family systems. Some of the themes that emerged and delineated the father’s role in parenting include- the father’s surface-level involvement with the children, the father’s cognizance of the importance of giving space to children, amongst others. The themes that were indicative of the mother’s role in parenting included- establishing healthy communication patterns, the mother’s active presence for children, authoritative style of parenting, amongst others. Although the differences between the mother’s and father’s style of parenting have been evident since long before the pandemic emerged, this unusual context did play a role in exacerbating these disparities. The findings are partially supported by a systematic review conducted by Yaffe (2023), in which he found that mothers are considered to be more accepting, supportive, and responsive, and are also more likely to make use of the authoritative style of parenting, as compared to the fathers. These findings can primarily be attributed to the social gender roles that are assigned to males and females, and the resultant gender stereotypes that affect virtually every domain of our social lives. For instance, traditional gender roles have assigned women the responsibility of indulging in household chores, while men are typically expected to financially support the family (Cerrato and Cifre, 2018).

The third global theme concerned itself with the changes in parenting practices as they occur over the generations, while also hinting at how certain practices are retained if found suitable for the present times. An intriguing finding from our study is that despite the evident differences in maternal versus paternal styles of parenting, there is indeed a shifting perception of fathers from the traditional role of a “distant guardian figure.” It is worth noting that these intergenerational influences on parenting exist irrespective of the unusual circumstances such as the Covid-19 pandemic. Madden et al. (2015) has found evidence of intergenerational transmission of parenting in a longitudinal study of 292 highly educated parents based in the UK. Similar results have been found in Eastern cultures as well, for instance, He et al. (2020), in their study on a sample of 192 participants from rural China found that the emotional warmth of one’s parents was significantly associated with caregiver’s warmth, whereas the rejective parenting style of one’s parents was consequently associated with the hostile parenting style of the caregiver toward their children. However, yet another study conducted in southern India with 10 families indicated that although warm parenting was transmitted across generations, the hostile parenting behavior was discontinued in the presence of external social support (Sekaran et al., 2021). Nonetheless, even though some researchers have initiated investigations of intergenerational transmission of parenting (for ex., Honig, 2014; Uba and Jain, 2019), there exists relatively marginal literature regarding such transmissions.

The fourth global theme outlined that parenting practices also hinge on the characteristics inherent to the children, such as their age, gender, birth order, etc. Whereas the pre-existing trends around child-centric parenting are well-documented in literature (Ashton-James et al., 2013), this type of parenting became especially relevant during the pandemic owing to the increased emotional needs of the children. Child-centred parenting seems to have become an indispensable feature of modern parenting but comes with its own set of repercussions. For instance, some researchers argue that centering parenting around one’s child can turn out to be detrimental to the parents’ well-being (Liedloff, 1975; Hodgkinson, 2009; Skenazy, 2009; Senior, 2010). However, yet another research has quashed such claims as the researchers found a positive correlation between child-centric parenting and parental well-being (Ashton-James et al., 2013). They argue in line with the pro-social investment hypothesis, stating that one’s personal well-being is associated with financial and emotional investment in others compared to oneself (Dunn et al., 2008).

The fifth and final global theme reflected on the context-dependent nature of parenting practices, as we found that the Covid-19 pandemic that disrupted every significant domain of our lives due to its devastating nature eventually provided parents with an opportunity to indulge in positive parenting practices, leading to evolved parent–child relationships. Evidently, the worldwide restrictions implemented owing to the pandemic resulted in changes in family structures globally (Menter et al., 2021), with India being no exception. However, many parents utilized that time to strengthen their family ties and develop friendly bonds with their children. This deeply resonated with a recent study conducted by Imran et al. (2021), which reported that about 92.7% (out of 330) of parents reported that they are spending more time with their children, with 67% reporting feeling very or extremely close to their children since lockdown. Therefore, the ‘quality time’ that the parents reported sharing with their children brought them closer together and led to evolved ‘parent–child dynamics’ in numerous cases as parents could reflect on their behavior and use those valuable insights to put them into practice during the pandemic.

Thus, the present study aptly describes the dynamic nature of parenting, as opposed to depicting it as a static entity, taking covid-19 as a context. While some parents initially grappled with adverse effects of pandemic-related stressors or resorted to avoidance strategies, others perceived pandemic as an opportunity to enhance their relationship with their children and gain deeper insights into their needs (Bülow et al., 2021). An interesting trend was also noticed, wherein pandemic-related stressors initially exerted a negative influence on parenting behavior. However, as the parents acclimated to the challenges hurled by the pandemic, a transformation in their parenting behavior from negative to positive trajectories was observed. This change within the same contextual framework further accentuates the dynamic and adaptive nature of parenting practices. In essence, the five themes emphasize the complex interplay between situational/contextual factors, child or parental characteristics, parental responses, and the evolving nature of parenting behavior.

There are numerous implications of this study. First and foremost, the study highlighted the evolving nature of parenting in response to changing situations and circumstances, though more research is needed to reflect on the functional aspect of it. For instance, whereas the dynamic nature of parenting can allow the parents to adjust to the ever-evolving needs of their children and navigating associated challenges, it may have unintended negative consequences in some situations. It is, therefore, imperative for parents to maintain an adequate balance between flexibility and consistency in their parenting strategies to ensure a positive outcome for their children. In addition, the data from the present study can be utilized by policymakers as well as officials to provide correct information to the larger population concerning how stressful situations and thereby stressors may affect family ties. For instance, it is critical that government should provide parents with knowledge of, for example, how the different age groups of children express distress and also the importance of sharing and talking about fears and negative emotions, at the time of distress (Dalton et al., 2020). Further, as the effects of the pandemic will possibly endure for some time and new pandemics may also arise in the future, the present study can inform mental health practitioners about the possible stressors as well as risk factors associated with a pandemic that are likely to influence the course of a parent–child relationship.

Although the current study presented us with an alternative perspective on the nature of parenting by exploring it in a novel context, the findings should be interpreted only in light of its limitations. As with all qualitative research, the present study also had a small sample size. Further, the sample consisted of participants from educated and fairly affluent families, and so, the findings may not be valid for the larger population of India. Future research should focus on including a more diverse sample belonging to varied socio-economic status and cultural backgrounds, to have greater conviction in our findings. Moreover, the present study attempted to understand the dynamics of parenting only through parents’ perspectives. Future research can also incorporate the adolescents’ perspectives so as to provide a more holistic understanding of the dynamic nature of parent-adolescent relationships which would enable the researchers to investigate the parents as well as adolescents’ effects in a single model, Again, the Covid-19 pandemic may have affected the parent–child relationship beyond the here studied time window. The researchers can follow up with the participants for a relatively longer duration of time in future research to have a more comprehensive understanding of the changing parent–child relationships. Finally, it is recommended that future researches utilize a mixed-model design to study this phenomenon, so that the results may be generalized to a larger population with a greater conviction.

## 5 Conclusion

In conclusion, this study explored the dynamic nature of parenting strategies. Our findings highlight the influence of stressors on parenting styles, emphasizing the responsiveness of parents to external factors. Moreover, we highlight gender differences in maternal and paternal parenting styles, stressing the importance of considering such dynamics in parenting research. Intergenerational changes in parenting practices are also explored, emphasizing the transmission of parenting behaviors across generations. Additionally, our study sheds light on the prevalence of child-centric parenting practices and their implications for family dynamics. Finally, amidst the COVID-19 pandemic, we observe the resilience and adaptability of families, as evidenced by the utilization of positive parenting practices.

## Data availability statement

The raw data supporting the conclusions of this article will be made available by the authors, without undue reservation.

## Ethics statement

The studies involving humans participants were approved by the Departmental Research Committee (DRC) of the Department of Psychology, University of Delhi, India. The studies were conducted in accordance with the local legislation and institutional requirements. The participants provided their written informed consent to participate in this study.

## Author contributions

NB: Conceptualization, Formal analysis, Methodology, Supervision, Validation, Writing – review & editing. MF: Conceptualization, Formal analysis, Methodology, Validation, Visualization, Writing – original draft. MA: Conceptualization, Data curation, Formal analysis, Investigation, Project administration, Writing – original draft.

## Appendix

**Semi-structured interview schedule**

o **Opening the interview – background information**
• Can you provide a brief background about yourself?
  *(Prompts related to their educational background, their life in general, and in recent times).*

o **Reflection on experiences in the pandemic**
• How has the pandemic been for you?
- Is there anything that you find particularly challenging?
  *(Prompts related to stressors faced, cope up strategies, etc.)*

- Is there anything that you found positive about this time?
  *(Prompts related to the utilization of time during the pandemic, familial ties, relationships with children, etc.)*

- How has your experience of parenting been during the pandemic?
- Is there anything that you find challenging about raising your children in these times?
  *(Prompts related to challenges faced in parenting an adolescent, how they deal with it, etc.)*

- Is there anything that you find positive about raising children during the pandemic?
  *(Prompts related to positive aspects of parenting in the pandemic, what they are particularly enjoying about it, etc.)*

o **Reflection on parenting practices**
• Can you tell me about your experience of being a parent?
  *(Prompts related to positive and negative aspects of being a parent)*

• How are your experiences of raising an adolescent?
- Is raising a child during adolescent years any different from raising them in early or middle childhood?
• Do you see any difference in raising a male or a female child? What, if any?
- What kind of strategies do you use in raising a female (and/or a male) child?
• Are there any differences in the way you have been raised by your parents and the way you are raising your children? Any similarities?
• How has the pandemic affected your relationship with your adolescent child?

o **Closing the interview**
• Now that we have talked at length about your experiences as a parent in general and during the pandemic, is there any other thing that you would like to add, which we may not have discussed but you think is relevant in this context?
• Do you have any questions that you would like me to answer?

So, these were the questions that I wanted to put to you. Thank you so much for taking out time and providing your valuable insights on this topic.
